# Application effect of PDCA circulation on nursing quality management and risk control in digestive endoscopy room

**DOI:** 10.1097/MD.0000000000035885

**Published:** 2023-12-01

**Authors:** Yan Xu, Chunhua Shi, Yun Liu

**Affiliations:** a Department of Gastraintestinal Endoscopy Room, Jingmen Central Hospital, Jingmen, Hubei, P.R. China; b Department of Respiratory, Jingmen Central Hospital, Jingmen, Hubei, P.R. China; c Gastroenterology Department, Jingmen Central Hospital, Jingmen, Hubei, P.R. China.

**Keywords:** digestive endoscopy room, nursing, PDCA cycle, quality assurance, risk management

## Abstract

To explore the application effect of plan, do, check, action (PDCA) cycle on nursing quality management and risk control in digestive endoscope room. Ninety patients who received digestive endoscopy care before undergoing PDCA circulation mode risk control from January 2022 to April 2022 were selected as the Common group. From May 2022 to December 2022, 156 patients who underwent digestive endoscopy care after undergoing PDCA cycle mode risk control were selected as the PDCA group. Compare the infection status of patients in the endoscope room and the qualification of the air in the endoscope room before and after PDCA circulation management. Compare the respiratory rate, heart rate, systolic blood pressure, diastolic blood pressure, and nursing satisfaction of patients in the Common group and the PDCA group. Compare the qualified rate of endoscopic cavity disinfection before and after PDCA cycle management, the qualified rate of endoscopic external disinfection, and the management score. Four patients in the Common group developed infection, with an infection rate of 4.44%. One case of infection occurred in the PDCA group, with an infection rate of 0.64%. The qualified rate of the endoscope room air in the Common group was 92.22%, while the qualified rate of the endoscope room air in the PDCA group was 98.72%. Compared with the Common group, the infection rate of patients in the PDCA group significantly decreased, and the qualified rate of air in the endoscope room significantly increased. The respiratory rate, heart rate, systolic blood pressure, diastolic blood pressure, nursing errors, and nursing complaint rates of patients in the PDCA group were significantly lower than those in the Common group, and nursing satisfaction was significantly higher than those in the Common group. The qualified rate of endoscopic cavity disinfection and endoscopic external disinfection in the PDCA group were significantly higher than those in the Common group. Compared with before management, the scores of post management, nursing safety, disinfection and isolation, instruments, theoretical tests, and operational tests of nursing personnel after management increased significantly. The PDCA cycle is well applied in nursing quality management and risk control in the digestive endoscope room.

## 1. Introduction

Digestive endoscopy is the most common method of diagnosis and treatment in digestive system diseases, and is widely used in clinical practice. Through examination, it can accurately grasp the patient’s condition, and provide certain guidance value for the subsequent clinical development of treatment plans, which is widely recognized by clinical and patient.^[1–3]^ Digestive endoscopy mainly combines chemical and electronic staining techniques to observe lesions, not only improving the clarity of observation, but also increasing the detection rate of digestive tract diseases, with a more prominent effect compared to traditional white light endoscopy.^[4,5]^ However, with the promotion and use of gastrointestinal endoscopy, it has been found in practical work that some patients are prone to trigger negative emotions such as tension, fear, and anxiety due to their low understanding of gastrointestinal endoscopy and significant physiological stress reactions, which directly hinder the development of examination work, and have a certain impact on the outcome of diagnosis and treatment, and may even increase the probability of risk events.^[6–8]^

After in-depth clinical research, some studies have found that providing corresponding nursing interventions during the implementation of digestive endoscopy can effectively prevent the occurrence of adverse events, while providing prerequisite guarantee for the smooth completion of diagnosis and treatment. However, the effect of conventional risk management in previous nursing was not ideal and targeted, making it difficult to achieve the effect of risk control.^[9–11]^ In recent years, with the continuous progress of medical technology and nursing level, it has been found that the plan, do, check, action (PDCA) cycle model has better effects in clinical practice. It mainly represents 4 stages of nursing work. As a whole cycle process, applying it to clinical nursing work can effectively prevent potential risks during nursing, protect patient privacy, increase patient satisfaction, and improve the integrity and service strength of nursing work.^[4,12,13]^

The purpose of this study was to explore the application effect of PDCA cycle on nursing quality management and risk control in the digestive endoscope room.

## 2. Materials and methods

### 2.1. Patients

The study was approved by the Ethics Committee of Jingmen Central Hospital. There were 90 patients who received digestive endoscopy care before undergoing PDCA cycle mode risk control from January 2022 to April 2022 as the Common group. There were 156 patients who underwent digestive endoscopy care after undergoing PDCA cycle mode risk control from May 2022 to December 2022 as the PDCA group. In the Common group, there were 39 male patients and 51 female patients, with an age range of 39 to 75 years. Gastroscopy was performed in 35 cases, colonoscopy in 30 cases, esophagoscopy in 21 cases, and duodenoscopy in 4 cases. In the PDCA group, there were 70 male patients and 86 female patients, with an age range of 37 to 76 years. Gastroscopy was performed in 76 cases, colonoscopy in 49 cases, esophagoscopy in 34 cases, and duodenoscopy in 18 cases.

### 2.2. Inclusion criteria

The patient and their family sign a letter of understanding. Patients can communicate normally. The patient met the indications for gastrointestinal endoscopy.

### 2.3. Exclusion criteria

Patients had organ dysfunction, mental disorders, tumor diseases, immune system diseases, and consciousness disorders. Patient was in poor compliance.

### 2.4. Management methods

Nursing intervention was carried out during the admission of patients in both groups for gastrointestinal endoscopy. The Common group carried out routine nursing management. Before digestive endoscopy, nursing personnel need to understand the patient’s past medical history and anesthesia history in detail, and fasting 8 hours before the examination, water deprivation, and enema 4 hours before the examination. During digestive endoscopy, their heart rate, respiration, and blood pressure should be observed, and any abnormalities found should be promptly reported to the physician.

Patients in the PDCA group underwent PDCA cycle mode management. Plan: a quality control team consisting of the director of the digestive department, head nurse, and nurse will be established to assess the risks in digestive endoscopy nursing in our hospital in accordance with the relevant regulations issued by the Ministry of Health. Based on the evaluation results, problems in digestive endoscopy nursing will be identified, and risk management objectives will be formulated. Failure to prepare according to medical instructions, poor standardization in various regions, lax verification, and inadequate management of bed fall are the primary risk issues in digestive endoscope nursing, and reasonable intervention plans need to be developed based on the risk issues. Provide systematic training for medical personnel to enhance their risk management awareness and nursing ability, continuously improve the hospital’s nursing management system during nursing, reasonably arrange various examination areas, and continuously organize, clean, and manage the digestive endoscope examination areas. Implementation: the hospital needs to conduct training and assessment on nursing theory, nursing operation, legal education, etc. for digestive endoscope nursing personnel every month according to the plan. Those who fail the assessment will be punished and retrained until they pass the assessment. Hospitals need to strengthen environmental management in digestive endoscopy departments, reasonably divide each area, and strictly follow the principle of sterility during the examination to avoid cross infection. In addition, items and equipment should be placed in appropriate locations to ensure reasonable handling. The hospital needs to divide positions according to the workload and difficulty of each position, so that each staff member can clarify their own job responsibilities, improve their sense of responsibility, and strengthen the effectiveness of risk management. Organize digestive endoscope nursing personnel to learn infection prevention and control rules and regulations, and strictly implement them in clinical nursing work to ensure nursing safety. The clinical use of sterile articles requires special personnel to manage and record them, and the hospital will regularly review and inspect them to ensure the quality and traceability of sterile articles. Examination: members of the quality control team regularly or irregularly inspect the quality of care. Treatment: summarize the existing problems in nursing based on the examination results, report the existing problems to the department, explore effective management methods, and supervise their implementation, so as to enter the next PDCA cycle.

### 2.5. Observation index

Record the infection status of the patient’s endoscope room, the eligibility of the air in the endoscope room, respiratory rate, heart rate, systolic blood pressure, diastolic blood pressure, nursing errors, nursing complaint rate, nursing satisfaction, the eligibility rate of endoscopic cavity disinfection, and the eligibility rate of endoscopic external disinfection.

To compare the risk management of instruments and devices, nursing safety, and first-aid drugs before and after the introduction of PDCA cycle mode in the department, the evaluation criteria were designed based on the 2011 version of the “Medical Quality Evaluation System and Assessment Standards.”^[14]^ There are 7 items, including post management, nursing safety, disinfection and isolation, emergency rescue drugs, instruments and devices, theoretical exams, and operational exams. The total score of each item is 100 points. The higher the score, the better the management effect.

### 2.6. Statistical analysis

All data in this study were statistically analyzed by SPSS 26.0 software (SPSS, Chicago, IL). *P* < .05 means the difference is statistically significant.

## 3. Results

### 3.1. Comparison of patient indoor air quality

Compare the infection status of the endoscope room and the qualification of the air in the endoscope room among the enrolled patients. Four patients in the Common group developed infection, with an infection rate of 4.44%. One case of infection occurred in the PDCA group, with an infection rate of 0.64%. The qualified rate of the endoscope room air in the Common group was 92.22%, while the qualified rate of the endoscope room air in the PDCA group was 98.72%. Compared with the Common group, the infection rate of patients in the PDCA group significantly decreased, and the qualified rate of air in the endoscope room significantly increased (Table 1).

**Table 1:** Comparison of air quality in patient inner diameter chambers.

Group	n	Number of infections	Air qualification rate
Common group	90	4 (4.44)	79 (87.78)
PDCA group	156	1 (0.64)	154 (98.72)
*X* ^2^		4.146	13.650
*P*		.042	.002

### 3.2. Comparison of patient physiological indicators

**Figure 1.**
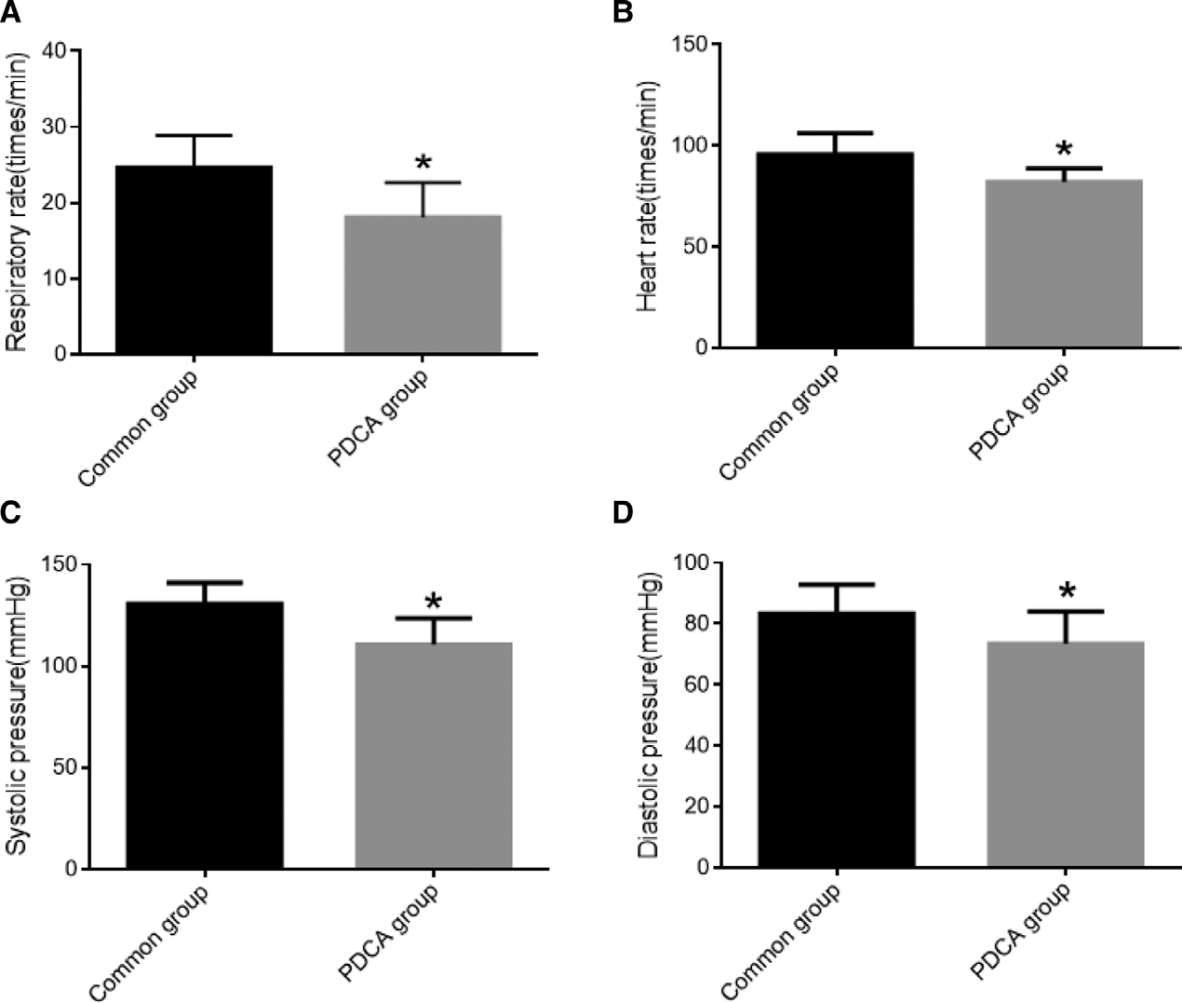
Comparison of physiological indicators such as respiratory rate (A), heart rate (B), systolic blood pressure (C), and diastolic blood pressure (D) in patients. *Note*: *, vs Common group, *P *< .05.

Compare the respiratory rate, heart rate, systolic blood pressure, and diastolic blood pressure of patients in the Common group and the PDCA group. The results showed that the respiratory rate, heart rate, systolic blood pressure, and diastolic blood pressure in the PDCA group were significantly lower than those in the Common group (Fig. 1).

### 3.3. Comparison of patient care satisfaction

We compared the nursing satisfaction findings of patients in the Common group and the PDCA group. After PDCA management, nursing errors and nursing complaint rates were significantly reduced, while nursing satisfaction was significantly increased (Table 2).

**Table 2:** Comparison of nursing satisfaction.

Group	n	Nursing errors	Nursing complaints	Nursing satisfaction
Common group	90	3	5	74
PDCA group	156	0	1	153
*X^2^*		5.264	5.793	4.484
*P*		.022	.016	.034

### 3.4. Comparison of detection of infection indicators in the endoscope room

The indicators of infection in the endoscope room include the qualified rate of disinfection of the endoscope cavity and the qualified rate of disinfection of the exterior of the endoscope. We conducted a total of 100 internal diameter inspections to compare the qualified rate of endoscopic cavity disinfection and the qualified rate of endoscopic appearance disinfection between the Common group and the PDCA group. We found that the qualified rate of endoscopic cavity disinfection and the qualified rate of endoscopic appearance disinfection in the PDCA group were significantly higher than those in the Common group (Table 3).

**Table 3:** Comparison of infection indicators in the endoscope room.

Group	n	Endoscopic cavity disinfection	Endoscopic appearance
Common group	50	48 (96.00)	47 (94.00)
PDCA group	100	100 (100.00)	100 (100)
*X^2^*		4.054	6.122
*P*		.044	.013

### 3.5. Comparison of patient management scores

**Figure 2.**
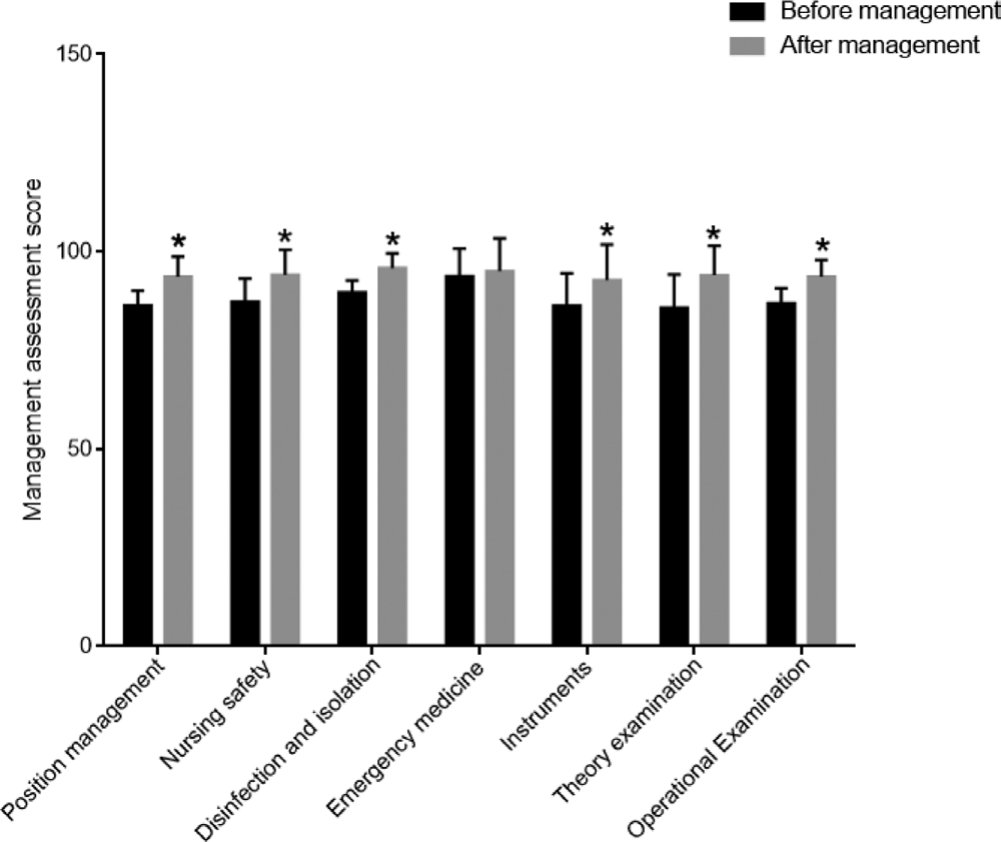
Comparison of management scores of nursing staff before and after PDCA cycle management. *Note*: *, vs Before management, *P *< .05.

We compared the management scores of 21 nursing staff enrolled. The management scoring items include post management, nursing safety, disinfection and isolation, first-aid drugs, instruments and devices, theoretical exams, and operational exams. Compared with before management, the scores of post management, nursing safety, disinfection and isolation, instruments, theoretical tests, and operational tests of nursing personnel after management were significantly increased (Fig. 2).

## 4. Discussion

The most important examination method for digestive system diseases is digestive endoscopy. In clinical practice, due to the need for endoscopy to be inserted from the patient’s anus, oral cavity, and other places, most of them are oral cavity insertion. During this process, it is extremely easy to cause obvious discomfort symptoms such as nausea, vomiting, and dizziness, which has a significant impact on the mental health and quality of life of patients.^[15–17]^ Moreover, due to the inherent operational reasons of this examination method, many patients’ feelings of pain during the examination lead to many problems with their actual situation.^[18–21]^

PDCA management mode is a new management concept proposed by a management expert in the United States, Dai Ming. It mainly includes 4 steps, namely, P (plan), D (implementation), C (inspection), and A (treatment). This mode forms a circular management process by formulating a management plan, implementing a plan, checking the implementation results, and formulating a new plan after dealing with problems in the management process, making the entire management mode more service intensive, more complete.^[22–24]^ When patients undergo endoscopic examination, they may experience certain discomfort and have a certain impact on their medical experience. The PDCA management model can effectively solve the above problems, optimize nursing plans, and improve nursing quality.^[25]^

Due to the special material of digestive endoscope, complete sterilization cannot be achieved before examination. Inserting digestive endoscope into the body can reduce the body’s defense ability and increase the risk of infection after examination, so it is necessary to strengthen risk management.^[26–28]^ With the development of current medical management models, PDCA cycle model management is gradually applied to clinical management, which can not only reduce nursing risks and nursing error events, but also improve nursing quality.^[29,30]^ During the implementation of the PDCA cycle model, first, evaluate the existing problems in nursing, develop reasonable intervention plans based on the evaluation results, target and address nursing problems through regular or irregular inspections, and then conduct PDCA management again to improve the risk management awareness of nursing personnel through a step-by-step model.^[31–33]^ From the results of this study, it can be seen that after the implementation of PDCA cycle management, the infection rate in the patient’s endoscope room significantly decreased, the air qualification rate in the endoscope room significantly increased, the patient’s respiratory rate, heart rate, systolic blood pressure, and diastolic blood pressure indicators significantly decreased, the rate of nursing errors and complaints significantly decreased, and nursing satisfaction significantly improved.

In addition, the PDCA cycle model can effectively ensure the development of diagnosis and treatment work. By emphasizing risk assessment and risk control work, such as standardized management and cleaning of digestive endoscope instruments, it can effectively ensure the safety of surgical treatment and avoid the occurrence of medical infections.^[34]^ In addition, the application of the PDCA cycle model can effectively strengthen the comprehensive quality of nursing personnel. By providing targeted training and assessment mechanisms, comprehensive skills can be improved, and the mastery of basic knowledge of digestive endoscopy and nursing professional level can be significantly improved.^[35]^ The results of this study also showed that nursing staff receiving PDCA management significantly increased their scores on job management, nursing safety, disinfection and isolation, instruments and devices, theoretical tests, and operational tests.

In conclusion, the application of PDCA cycle in nursing quality management and risk control in digestive endoscope rooms has a good effect and is worthy of wide promotion.

## Author contributions

**Conceptualization:** Yan Xu, Chunhua Shi, Yun Liu.

**Data curation:** Yan Xu, Chunhua Shi.

**Formal analysis:** Yan Xu, Chunhua Shi.

**Investigation:** Yan Xu, Chunhua Shi, Yun Liu.

**Methodology:** Yan Xu, Chunhua Shi, Yun Liu.

**Software:** Yun Liu.

**Supervision:** Chunhua Shi.

**Writing – original draft:** Yan Xu, Chunhua Shi.

**Writing – review & editing:** Yan Xu, Chunhua Shi, Yun Liu.
